# ID 227 - Eficácia e Segurança do Dupilumabe em Comparação ao Omalizumabe para Asma Grave com Fenótipo Alérgico: revisão sistemática com metanálises de comparações indiretas

**DOI:** 10.5327/2237-9622.2025.v34s1.227

**Published:** 2025-11-25

**Authors:** Aline Garcia Barbosa, Frederico Magro, Greice Buttelli, Renata Prioli, Alexandre Taminato, Sarah Franco Watanabe, Bianca Salvador, Bruna Marmett, Celina Borges Migliavaca, Gilson Dornelles, Nayê Balzan Schneider, Maicon Falavigna

**Keywords:** asma, imunoterapia, revisão sistemática, metanálise em rede

## Abstract

**Introdução:**

A asma é caracterizada por inflamação crônica das vias aéreas. Entre seus fenótipos e endotipos, a inflamação tipo 2 (fenótipos alérgico e eosinofílico) é caracterizada por manifestação precoce e mais grave. Atualmente, o omalizumabe (OMA) é o único imunobiológico disponível no Sistema Único de Saúde (SUS) para o tratamento da asma grave com fenótipo alérgico. Recentemente, o dupilumabe (DUPI) – um anticorpo monoclonal humano antagonista do receptor da IL-4 e da IL-13 – surgiu como alternativa para essa condição.

**Método:**

Foram conduzidas duas revisões sistemáticas avaliando a efetividade do DUPI e do OMA em relação ao placebo no tratamento da asma grave com fenótipo alérgico, definido como IgE ≥ 30 UI/mL e sensibilidade a um ou mais aeroalérgenos perenes. Apenas estudos clínicos randomizados, com mascaramento de participantes e avaliadores, foram incluídos, devido ao componente subjetivo na definição de exacerbação. Também foram incluídos apenas estudos que avaliavam a adição de OMA ou DUPI ao tratamento com beta-agonistas de ação prolongada e corticosteroides inalatórios, conforme PCDT de asma. As buscas foram realizadas nas bases MEDLINE (PubMed), EMBASE e Cochrane CENTRAL, em janeiro de 2024. O risco de viés dos estudos individuais foi avaliado com o instrumento Risk of Bias 2.0, com a certeza da evidência sendo avaliada com o sistema GRADE (do inglês, ). A partir dos resultados identificados nas duas revisões independentes, foi realizada metánalise de comparações indiretas. As análises foram feitas com o software R, pacotes meta e netmeta, utilizando abordagem frequentista.

**Resultados:**

Quatro ensaios clínicos avaliaram DUPI e oito avaliaram OMA. Em comparação ao placebo, DUPI reduziu o risco de exacerbações em 52% (RR 0,48; IC 95% 0,37 a 0,62; alta certeza da evidência) e aumentou o VEF1 em 0,16 L (IC 95% 0,12 a 0,20 L; alta certeza da evidência), além de melhorar a qualidade de vida e o controle da doença. OMA, em comparação ao placebo, reduziu as exacerbações em 26% (RR 0,74; IC 95% 0,64 a 0,86; baixa certeza da evidência por risco de viés e evidência indireta) e aumentou o VEF1 em 0,08 L (IC 95% 0,04 a 0,11 L; baixa certeza por risco de viés e evidência indireta). Comparação indireta mostrou que DUPI reduz a taxa de exacerbações em 32% em relação ao OMA (RR 0,68; IC 95% 0,54 a 0,88; baixa certeza da evidência por risco de viés e evidência indireta em OMA) e aumenta o VEF1 em 0,08 L (IC 95% 0,03 a 0,13; p = 0,001; baixa certeza por risco de viés e evidência indireta em OMA). No desfecho de descontinuação por eventos adversos, não houve diferença estatística entre os tratamentos, embora tenha sido observada redução de 26% com o DUPI comparado ao OMA (RR 0,74; IC 95% 0,28 a 1,93; moderada certeza da evidência por imprecisão).

**Conclusão:**

OMA e DUPI são eficazes no tratamento da asma grave com fenótipo alérgico, com a incorporação do OMA pelo SUS, em 2019, sendo um marco importante para a atenção ao paciente com asma grave no SUS. A comparação indireta indica que DUPI está provavelmente associado a uma menor taxa de exacerbações e a uma melhora da função pulmonar em relação ao OMA, tornando-se uma opção viável para inclusão no SUS. Apesar dos resultados promissores, é essencial contextualizar o impacto clínico com aspectos econômicos para garantir a sustentabilidade do sistema com a adoção de tratamentos custo-efetivos.

